# A traditional Chinese herbal formula improves pressure ulcers in paraplegic patients: A randomized, parallel-group, retrospective trial

**DOI:** 10.3892/etm.2013.1053

**Published:** 2013-04-09

**Authors:** XIN LIU, QINGXI MENG, HUA SONG, TINGBAO ZHAO

**Affiliations:** 1 Department of Spinal Cord Injury, Institute of Orthopedics and Traumatology of Chinese PLA, General Hospital of Jinan Military Area Command, Jinan, Shandong 250000;; 2 Department of Urology, The Second Hospital of Shandong University, Jinan, Shandong 250033, P.R. China

**Keywords:** pressure ulcer, stage IV, traditional Chinese herbal formula, paraplegic patients, nursing

## Abstract

In this study, the efficacy of a novel Chinese herbal formula, cure rot and flat sore ointment (CRFSO), in the management of stage IV pressure ulcers, and the effect of simultaneous comprehensive rehabilitation in improving the outcome were evaluated. A total of 35 paraplegic patients with stage IV pressure ulcers who underwent reconstruction and inpatient rehabilitation from January 2004 to September 2010 were included in the study. *Arnebia* root oil (ARO) was used on 16 patients with 11 ulcers (stage IV). The remaining 19 patients with 20 ulcers (stage IV) received a traditional Chinese herbal formula (CRFSO). After 28 days of treatment, the wound healing results, in particular, the healing rate, effectiveness rate, improvement rate and no response rate were evaluated. Six patients from the ARO group sought other types of therapy due to their own consideration of poor efficacy. After 28 days of treatment, the wound healing result and no response rate demonstrated a statistically significant difference (P<0.005) between the two groups, suggesting that the novel Chinese herbal formula is an effective treatment for pressure sores in paraplegic patients. All outcome variables demonstrated significant improvement in the CRFSO group compared with the ARO group after 28 days of treatment, with a higher healing rate (85% in the CRFSO group and 45.45% in the ARO group) and lower no response rate (5% in the CRFSO group and 18.18% in the ARO group). The traditional Chinese herbal formula improved pressure sores in paraplegic patients effectively and inpatient rehabilitation was also significantly improved.

## Introduction

Pressure ulcers are caused by long-term compression of parts of the body, blood circulation disorder, sustained ischemia, hypoxia of local tissue and nutritional deficiencies. This leads to the loss of normal function of skin and causes tissue damage and necrosis (1). The pressure leads to poor circulation and eventually contributes to cell death, skin breakdown and the development of an open wound. If not adequately treated, open ulcers become a source of pain, disability and infection. The prevalence of pressure ulcers in nursing home residents ranges from 8 to 24% and the annual incidence approaches 12% (2–4). Methods to prevent and control pressure ulcers are the main focus of clinical nursing (5). The standard options for treating pressure ulcers include surgical, mechanical, autolytic and enzymatic debridement (6). In China, therapies collectively called traditional Chinese medicine (TCM) are commonly used. This includes cured rot and flat sore ointment (CHMO), acupuncture and moxibustion (7). TCM has been used for the prevention and treatment of ulcers for many years. A variety of methods have been used for pressure ulcer treatment, although they have different prescription formulations, the role of the main approach is to improve local blood circulation and enhance tissue regeneration (8).

The doctors at The General Hospital of Jinan Military Area Command (Jinan, China) use a variety of drugs for the treatment of pressure ulcers, including *Arnebia* root oil (ARO), Jing Wan Hong ointment and YuHong ointment. However, none of these effectively alleviate the pain of patients lying in bed and they have a slow curative effect and long healing time. With years of clinical experience of the treatment of pressure ulcers and TCM research, we developed a TCM ointment called cured rot and flat sore ointment (CRFSO), composed of gypsum fibrosum (18 g) and three herbal medicines: hydrargyrum oxydatum crudum (9 g); red orpiment (6 g) and borneol (0.9 g). Following clinical use, the effect of CRFSO in the treatment of pressure sores was observed to be superior to that of other drugs. For verification, a randomized, controlled and retrospective clinical study was conducted to evaluate the efficacy and safety of CRFSO. A total of 35 consecutive patients with pressure sores following paraplegia received CRFSO or ARO treatment from January 2004 to September 2010. The results of our study demonstrate that compared with ARO, our novel Chinese herbal formula relieved pressure ulcers in paraplegic patients with a shorter healing time and clear curative effect.

## Materials and methods

### Materials

ARO was purchased from Xi’an Realherb Biotech Co., Ltd. (Xi’an, China) and CRFSO was prepared by The General Hospital of Jinan Military Area Command.

### Patients

This observational study was conducted from January 2004 to September 2010. The pressure ulcers were classified into four stages (I, II, III and IV) according the guidelines of the National Pressure Ulcer Advisory Panel. The pressure ulcer treatment information of 35 paraplegic patients (69 ulcers) caused by spinal cord injury from January 2004 to September 2010 was reviewed. The data were collected from patient records in The General Hospital of Jinan Military Area Command. Written consent for data and image use from all patients was received preoperatively. The inclusion criterion was at least one pressure ulcer in stage IV. Patients with ulcers from other causes or a primary site of pathology other than paraplegia were excluded. Socio-demographic and clinical data were obtained in a predesigned format. Socio-demographic information, including age, gender and primary illness was obtained prior to treatment.

After treatment for 2 weeks, 6 patients sought other modes of therapy, including surgical methods or CRFSO, due to their own consideration of poor efficacy; these were all from the ARO group. The other 29 patients completed the treatment evaluation successfully. No statistical differences in the two groups of patients were identified in ratio of gender, mean age and mean area of pressure ulcer in different stages. The 29 patients in the two groups had a total of 57 ulcers (31 were stage IV and 26 under stage IV). Table I shows the location distribution of these pressure ulcers. ARO was applied to 10 patients with a total of 20 ulcers (11 ulcers at stage IV) and the other 19 patients with a total of 37 ulcers (20 ulcers at stage IV) received the novel Chinese herbal formula. The demographic data (Table II) demonstrated no significant differences (P>0.05). The study was approved and registered by The General Hospital of Jinan Military Area Command in March 2010, the Ethics committee approved the screening, treatment and data collection of these patients. All subjects signed the written informed consent form. All work was undertaken following the provisions of the Declaration of Helsinki.

### Procedures

A 0.9% sodium chloride injection was used to clean wounds (ulcers) and then vesicular, ulcerated and necrotic tissue was removed. An infrared lamp was used to irradiate the disinfected pressure ulcers and the surrounding 2–3 cm area for ∼30 min. Following routine disinfection, the majority of the necrotic flesh was cut away using repeated hydrogen peroxide and saline cleaning. Then the ARO or CRFSO was administered to the affected area.

The ARO group was treated with the application of a gentamicin wet gauze (100 ml 0.9% sodium chloride injection plus 240,000 units gentamicin) on the pressure ulcers and the CRFSO group received 68 g CRFSO. Aseptic dressing and bandages were used in the two groups and changed every 1–2 days until the ulcers had healed. All patients underwent necessary care and pain treatment during the study.

### Evaluation methods

Evaluation was conducted after 28 days of treatment. The therapeutic effect was divided into the following grades: i) healing: the ulcer healed, became a scab and was shed; ii) effective: the ulcer was apparently contractible and the growth of granulation tissue was good with significant alleviation of pain; iii) improved: the ulcer area and secretion were reduced and pain was alleviated; and iv) no response: no changes or the local wound was infected. The therapeutic duration was recorded as the ulcer healing time.

### Statistical analysis

Therapeutic duration is presented as mean ± SD. Comparisons of continuous variables between the two groups were performed by one-way analysis of variance (ANOVA). If the F distribution was significant, a t-test was used to specify differences between groups. P<0.05 was considered to indicate a statistically significant difference. The SPSS 19.0 software package (SPSS, Inc., Chicago, IL, USA) was used for the statistical tests.

## Results

**Figure 1:**
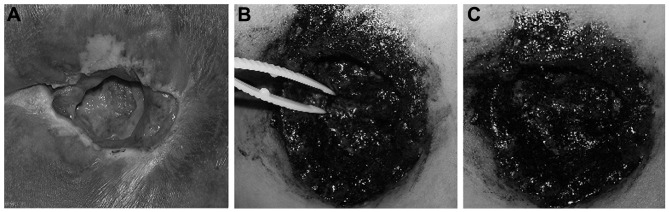
Macroscopic observation results of pressure ulcers (stage IV) with cure rot and flat sore (CRFS) ointment (A) before treatment; (B) 2 weeks after treatment, scab healing; and (C) 3 weeks after treatment, scab almost healed and shed.

**Figure 2:**
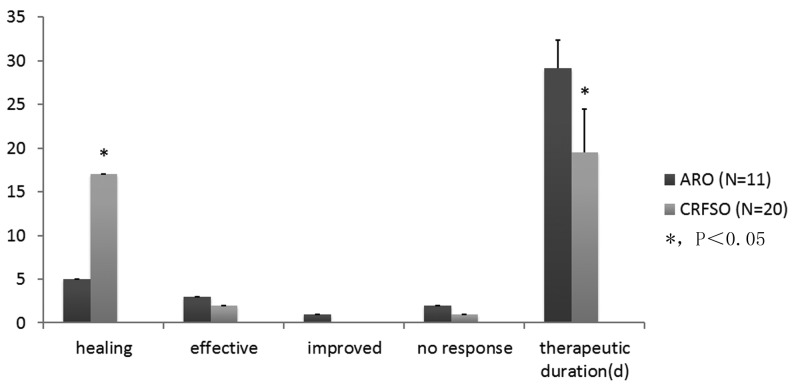
Therapeutic effect and duration comparison in the cure rot flat sore ointment (CRFSO) and *Arnebia* root oil (ARO) groups.

No patient had any ointment-related serious adverse reaction during the treatment. Of the 29 patients that received external application of ointment to treat pressure ulcers, we observed a higher healing rate (17 of 20 in the CRFSO group and 5 of 11 in the ARO group) and lower no response rate (1 of 20 in the CRFSO group and 2 of 11 in the ARO group) in patients treated with CRFSO compared with ARO. Treatment was effective in 2 of 20 patients in the CRFSO group and 3 of 11 in the ARO group. Improved results were observed in 1 of 11 ulcers in the ARO group. Fig. 1 shows the treatment result of one pressure ulcer (stage IV) with CRFSO. The therapeutic duration of treatment with ARO ranged from 19 to 43 days (mean, 29.18±3.18 days); a relatively shorter treatment time was recorded for CRFSO, which ranged from 14 to 36 days (mean, 19.47±4.99 days). The therapeutic effect and therapeutic duration of the two groups are summarized in Fig. 2. Statistical analysis revealed that the therapeutic duration of the two groups was significantly different (P<0.05).

Compared with ARO, the CRFSO treatment time is shorter and the patient outcome is improved. This traditional Chinese herbal formula relieves pressure sores in paraplegic patients effectively and inpatient rehabilitation is significantly improved.

## Discussion

Pressure ulcer management is divided into non-surgical and surgical methods. Drug-based inhibition of pressure ulcers by the use of a single medicine for external application, has provided a useful and cost-effective procedure without risk to the patient (9). A pressure ulcer in paraplegic patients is a unique type of chronic ulcer with its own characteristics and healing is much more difficult compared with that of an ordinary wound. Despite aggressive treatment for pressure ulcers, individualized therapy must be tailored to each patient according to gender, age, pathophysiology, expectations and financial situation.

TCM and its extracts are commonly used topically in the clinic and show unique efficacy, particularly for burns, diabetic foot ulcers, cervical erosion, herpes zoster, chronic ulcers (oral and skin) and warts (10–13). We developed a TCM ointment, (CRFSO), which comprises four ingredients: gypsum fibrosum, hydrargyrum oxydatum crudum, red orpiment and borneol. Gypsum fibrosum was used to dilute the concentration of the active organic ingredient (14) and to treat heat syndrome according to the ‘Yin-yang’ theory (15). Hydrargyrum oxydatum crudum is mainly used in the treatment of furuncles, trauma and anal fistulae (16). Red orpiment (Realgar) is an important component of traditional Chinese medicine formulation, which have effects of detoxifying, insecticidal and drying (17). Borneol is commonly used on the skin to accelerate the permeability of other pharmaceutical ingredients (18). Combined with other traditional Chinese formulae, we identified a formulation with reasonable proportions. During years of clinical application, this formula has been shown to be effective.

Compared with Western medicine, the toxicity and adverse effects of Chinese medicines are fewer and relatively more common in meridians and collaterals (19). According to of Chinese herbalism theory, interactions among the herbs may produce synergistic effects and neutralize the potential toxicity or side-effects of the individual constituents (20). A number of clinical studies have demonstrated the efficacy of TCM in the treatment of pressure ulcers (12,13,20).

In our retrospective evaluation of the efficacy of CRFSO in the treatment of stage IV pressure ulcers, the average healing rates in the ARO and CRFSO groups were 45.45% and 85%, respectively. A shorter therapeutic duration was recorded in CRFSO group compared with ARO group; average 29.18±3.18 days in the ARO group and 19.47±4.99 days in the CRFSO group.

The TCM principle of ‘boosting qi, increasing collateral dredging, activating stagnant blood and dissolving stasis’ (21–24) was applied in our formula and the positive outcome of this study demonstrated that the TCM formula CRFSO is a promising treatment for pressure ulcers.

In recent years, combinations of traditional Chinese and Western medicine treatment have been used as effective clinical treatments for a number of diseases (25,26). With the result of the current retrospective clinical evaluation, further studies concerning the possibility of combing this novel Chinese herbal formula with Western medicine to treat pressure ulcers is required. Additionally, limited by sample size, the results require further confirmation in a larger randomized, controlled clinical trial, which is ongoing.

## Figures and Tables

**Table I:** Site of pressure ulcers in paraplegic patients.

Site of ulcers	No. of patients	No. of ulcers	No. of stage IV ulcers
Sacrococcygeal region	11	24	16
Hip	11	19	8
Ankle	5	10	5
Heel	2	4	2

**Table II:** Baseline characteristics of the patients (n=29).

Characteristics	ARO group	CRFSO group	P-value
No. of patients	10	19	>0.05
No. of male patients	6	13	>0.05
Median age (years)	58. 6±3.5	59.7±4.2	>0.05
Number of ulcers at stage IV	11	20	>0.05
Pressure ulcer area (cm^2^)	14.8±2.6	16.1±3.3	>0.05

ARO, *Arnebia* root oil; CRFSO, cure rot and flat sore ointment.
